# Tension pneumoperitoneum after surgery for endometrial cancer and hernia in a morbidly obese female: a case report

**DOI:** 10.1186/s13256-018-1581-7

**Published:** 2018-03-20

**Authors:** Bing-Sheng Lin, Yan-Shen Shan, Wan-Chen Liu, Chin-Han Wu, Pei-Ying Wu, Keng-Fu Hsu

**Affiliations:** 1 Department of Family Medicine, National Cheng Kung University Hospital, College of Medicine, National Cheng Kung University, Tainan, Taiwan; 2 Department of Surgery, National Cheng Kung University Hospital, College of Medicine, National Cheng Kung University, Tainan, Taiwan; 3 Department of Radiology, National Cheng Kung University Hospital, College of Medicine, National Cheng Kung University, Tainan, Taiwan; 4 Department of Obstetrics and Gynecology, National Cheng Kung University Hospital, College of Medicine, National Cheng Kung University, 138 Sheng Li Road, Tainan, 704 Taiwan

**Keywords:** Tension pneumoperitoneum, Endometrial cancer, Hernia, Obesity

## Abstract

**Background:**

Obesity is a risk factor for the development of endometrial cancer and abdominal wall hernias. We report a case of tension pneumoperitoneum that developed after gynecological surgery and mesh repair of a ventral hernia.

**Case presentation:**

A 57-year-old Asian Taiwanese woman with a body mass index of 52.9 (kg/m^2^) underwent total abdominal hysterectomy and bilateral salpingo-oophorectomy due to endometrial cancer, and ventral herniorrhaphy with mesh due to ventral hernia. Tension pneumoperitoneum with severe dyspnea developed on postoperative day 14. Rather than performing emergency laparotomy as in visceral perforation, a transabdominal catheter was inserted to drain the intra-abdominal gas. This approach dramatically relieved the tension pneumoperitoneum and dyspnea. Our patient then recovered smoothly; the catheter was removed on postoperative 24, and she was discharged on postoperative day 28. The clinical course of the endometrial cancer and repaired ventral hernia was well at the 1-year follow-up.

**Conclusions:**

Tension pneumoperitoneum, which may result from the valve effect of unhealed abdominal mesh, could develop after gynecological surgery and hernia mesh repair in obese patients. Under these conditions, emergency drainage of the intra-abdominal gas by catheter insertion is sufficient to relieve the abdominal pressure and correct the conditions, while emergency laparotomy as in visceral perforation is unnecessary and may increase patient morbidities.

## Background

A high body mass index (BMI) is an established risk factor for the development of endometrial cancer [1]. Obesity predisposes patients to abdominal wall hernias. Obese patients with a higher BMI undergoing abdominal wall repair often have increased complications, including abdominal compartment syndrome, seroma, hematoma, infectious morbidity, and dehiscence, which typically occur early in the postoperative period [2, 3].

The accumulation of free intra-abdominal air with elevated intra-abdominal pressure is known as tension pneumoperitoneum (TP). TP may result in an elevation of the diaphragm, which reduces lung volume and compresses the inferior vena cava, which in turn reduces the venous return and cardiac output and can result in aortic occlusion [4]. Various etiologies have been reported, including gastrointestinal perforation, barotrauma in ventilated patients, and diaphragmatic hernia repair [5]. Here, we report the case of a morbidly obese woman with TP that developed after surgery for endometrial cancer and the mesh repair of a ventral hernia. Rather than performing emergency laparotomy as in visceral perforation, she received drainage of the intra-abdominal gas by a transabdominal catheter which relieved the TP and her dyspnea.

## Case presentation

**Fig. 1:**
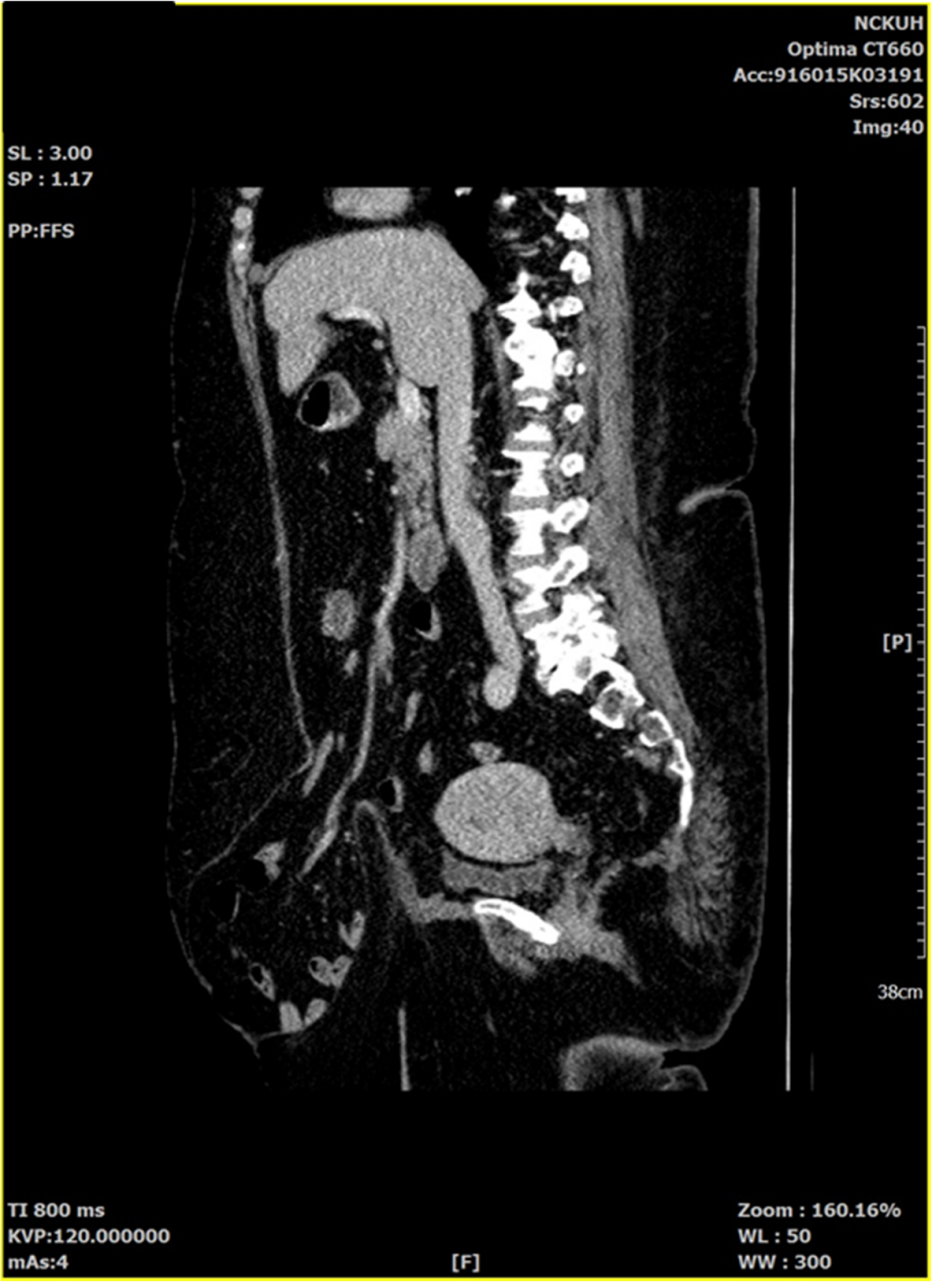
Computed tomographic image showing a large ventral hernia sac

A 57-year-old gravida 6, para 3, Asian Taiwanese woman presented to our hospital for scheduled surgery due to endometrial cancer. At admission, her body temperature was 36.8 °C, pulse rate 78 beats/minute, respiratory rate 20/minute, and blood pressure 136/98 mmHg. Her family and environmental history were unremarkable. She did not receive surgery before nor take any medication for systemic disease, such as diabetes mellitus or hypertension. She was a housekeeper. She did not smoke tobacco or consume alcohol. On physical examination, she had normal breathing sound, without wheezing or crackle, and a regular heartbeat without murmur. Her abdomen was soft with normoactive bowel sound on auscultation. Her extremities were freely moveable without edema. She exhibited severe morbid obesity (body weight, 128.4 kg; height, 155.7 cm; BMI, 52.9 kg/m^2^). Her daily activities were independent most of the time, except for some episodes of exertional dyspnea. Computed tomography (CT) revealed a ventral hernia with a large fascial defect (Fig. 1). There were no clinical symptoms, such as abdominal pain or tenderness. She underwent total abdominal hysterectomy, bilateral salpingo-oophorectomy, and ventral herniorrhaphy with Goretex mesh (GORE DUALMESH®).

**Fig. 2:**
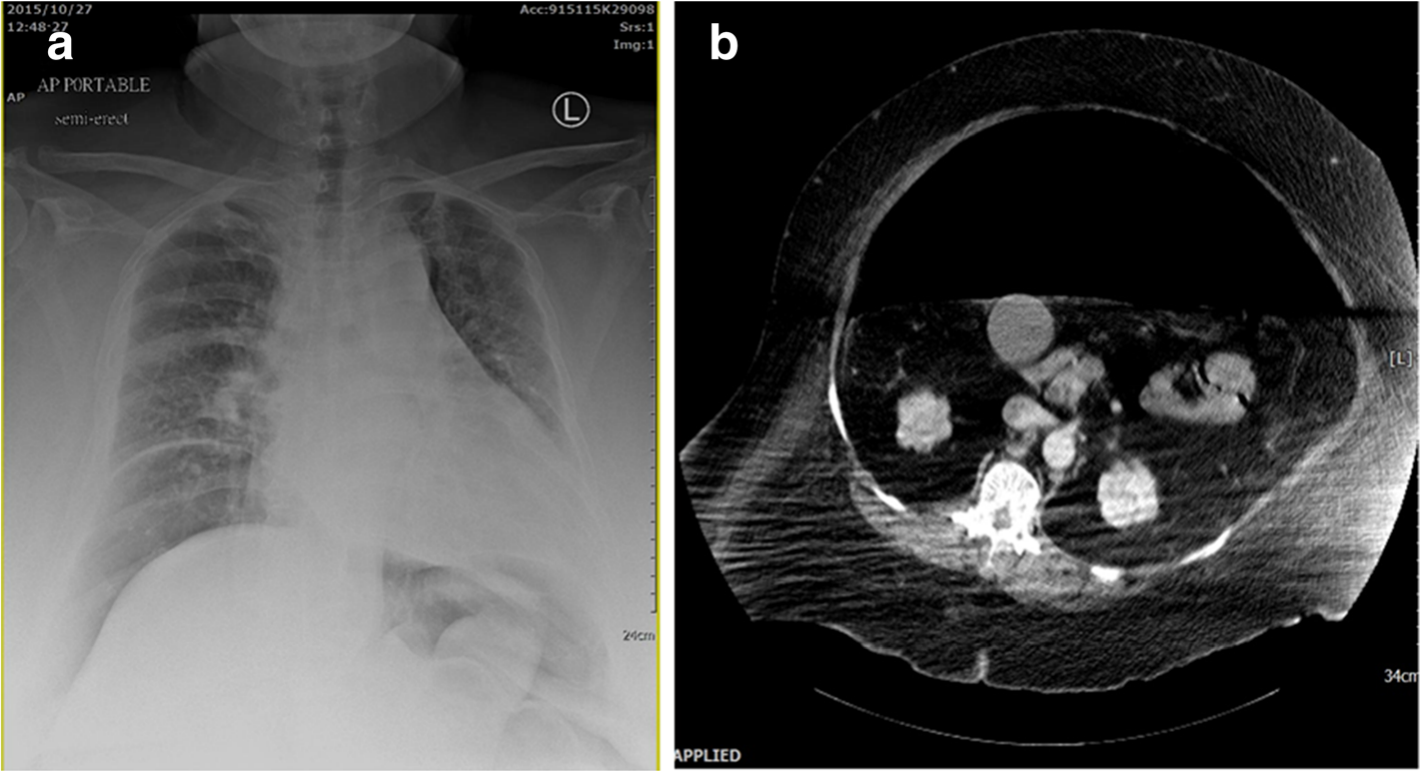
**a** Chest X-ray demonstrating an elevated diaphragm and suspicious pneumoperitoneum. **b** Computed tomography scan, transverse view. Massive free air and minimal ascites were noted in the abdomen

Her early postoperative course was smooth. She consumed water on postoperative day (POD) 3, a clear-liquid diet on POD5, and a semi-liquid diet on POD7. She began bedside ambulation, and half of her stitches were removed on POD10. However, progressive dyspnea was noted after bedside ambulation. Intra-abdominal free air with elevation of right-side hemidiaphragm was noted by chest X-ray (Fig. 2a) on POD14. There was no abdominal tenderness or rebounding pain, but there was moderate abdominal distension. She was kept under close observation and began nil by mouth (NPO) for possible visceral perforation.

Two days later (POD16), severe dyspnea and extensive abdominal distention were noted. Her respiratory rate was elevated to 26/minute with accessory muscle use. Arterial blood gas analysis (under simple O_2_ mask, 10 L/minute) showed: pH, 7.42; partial pressure of carbon dioxide in arterial blood (PaCO_2_), 44.8 (mmHg); partial pressure of oxygen in arterial blood (PaO_2_), 86.2 (mmHg); bicarbonate (HCO_3_-), 28.5 (mmol/L); blood oxygen saturation (SpO_2_), 96.7%; and blood pressure, 102/75 mmHg. Laboratory tests revealed leukocytosis with a white blood cell (WBC) count of 12,900/uL, hemoglobin (Hb) 12.6 g/dL, and platelet count 310,000/uL. Her C-reactive protein (CRP) was elevated at 165.7 mg/dL. Serum creatinine was 0.99 mg/dL, while serum sodium was 131 mmol/L, and potassium was 3.9 mmol/l. A physical examination showed severe abdominal distension without rebound pain. A pelvic examination demonstrated good healing of the vaginal cuff without leakage. CT showed a large amount of intra-peritoneal free air with little ascites (Fig. 2b). Because clinical visceral perforation was less likely, under the impression of pneumoperitoneum, in this emergent situation, an 8-Fr. pigtail catheter was inserted under CT guidance for decompression. A massive amount of gas was drained. Subsequently, her dyspnea and abdominal distention dramatically subsided. She re-started oral intake on POD20, and the abdominal pigtail catheter was removed on POD24 after no more free air was revealed by chest X-ray. No further abdominal distention was noted, and she was discharged on POD28. The clinical course of the endometrial cancer and repaired hernia were well at the 1-year follow-up.

## Discussion

In our case, the TP may have resulted from the valve effect of unhealed hernia mesh. Unlike the TP caused by gastrointestinal perforation which needs emergency laparotomy to repair the perforation, it was unnecessary for our patient. The drainage of intra-abdominal free air is sufficient to improve this condition. The most common iatrogenic cause of pneumoperitoneum is abdominal surgery. Postoperative pneumoperitoneum is usually absorbed within 2 weeks. When pneumoperitoneum progresses with increasing intra-abdominal pressure, hemodynamic and ventilatory compromise might occur, and the condition results in TP. Dyspnea and venous congestion in the lower extremities might be noted during the physical examination. Similar to tension pneumothorax, emergency percutaneous needle decompression is needed [6]. When managing hollow organ perforation-related TP, a venous catheter may be percutaneously inserted to stabilize the patient’s vital signs and to bridge the time to the start of the emergency operation [5].

**Fig. 3:**
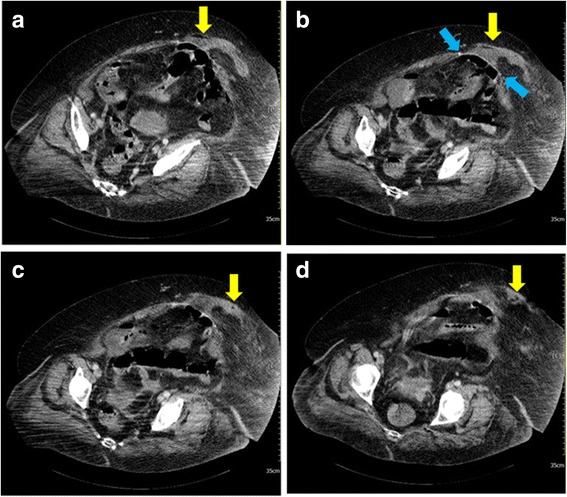
Computed tomography scan, contrast-enhanced transverse view, cranial (**a**) to caudal (**d**). Series of subcutaneous fluid accumulation (*yellow arrows*) extending from the mesh (between *blue arrows*) to the superficial incision wound

Ventral hernia mesh repair may reduce tension on the abdominal wall, resulting in less recurrence compared with a simple suture repair. Various complications associated with hernia repair have been reported [2, 3]. However, there are few reports in the literature regarding TP as a complication related to hernia repair. In our case, abdominal free air was noted on POD14 after ambulation. The most plausible cause of TP was that air may have entered our patient’s abdominal cavity via the unhealed abdominal wound, while the one-way valve effect developed due to morbid obesity. When reviewing the series of CT scans done on POD16, we found that fluid had accumulated from the mesh extending to the superficial wound (Fig. 3a–d), indicating the formation of a tract that allowed air to enter her abdominal cavity. When our patient began to ambulate, the thick abdominal subcutaneous fat and muscle detached from the mesh. This effect may have caused the formation of a small tract extending from the superficial wound to the subcutaneous fat and mesh and into her abdominal cavity. When she exhaled, an upward movement of her diaphragm created negative pressure and caused a small amount of gas to enter her abdominal cavity. When she inhaled, although the downward movement of her diaphragm created a relative positive intra-abdominal pressure, her bowel may have sealed the tract, resulting in the prevention of free air escaping from her abdominal cavity. When she was lying down, the subcutaneous fat and muscle re-attached to the mesh, preventing the free air from leaving her abdominal cavity. Due to the valve effect, air accumulated gradually over several days and finally developed into TP.

Unlike the TP caused by gastrointestinal perforation, it was unnecessary for our patient to receive emergency laparotomy to repair the perforation. The drainage of intra-abdominal free air is sufficient to improve this condition. As the abdominal wound healed, the gas route sealed gradually.

## Conclusions

Most surgery-related cases of pneumoperitoneum resolve spontaneously within 2 weeks after surgery. Caution should be taken if the pneumoperitoneum occurs or progresses 2 weeks after surgery in an obese patient with a mesh-repaired hernia. If accompanied by dyspnea, abdominal distention, and hemodynamic change, the possibility of TP should be considered, and emergency drainage of the intra-abdominal gas is needed. Emergency laparotomy is unnecessary for this condition.

## Abbreviations

BMI: Body mass index
CT: Computed tomography
POD: Postoperative day
TP: Tension pneumoperitoneum
